# The effect of apigenin, an aryl hydrocarbon receptor antagonist, in Phthalate‐Exacerbated eosinophilic asthma model

**DOI:** 10.1111/jcmm.17804

**Published:** 2023-06-14

**Authors:** Seo‐Hee Kim, Quang Luu Quoc, Hae‐Sim Park, Yoo Seob Shin

**Affiliations:** ^1^ Department of Biomedical Science Graduate School of Ajou University Suwon‐si South Korea; ^2^ Department of Allergy and Clinical Immunology Ajou University School of Medicine Suwon‐si South Korea

**Keywords:** asthma, eosinophil, epithelium, phthalate, T cell

## Abstract

Endocrine disrupting chemicals have been known to contribute to the aggravation of inflammatory diseases including asthma. We aimed to investigate the effects of mono‐n‐butyl phthalate (MnBP) which is one of the representing phthalates, and its antagonist in an eosinophilic asthma mouse model. BALB/c mice were sensitized by intraperitoneal injection of ovalbumin (OVA) with alum and followed by three nebulized OVA challenges. MnBP was administered through drinking water administration throughout the study period, and its antagonist, apigenin, was orally treated for 14 days before OVA challenges. Mice were assessed for airway hyperresponsiveness (AHR), differential cell count and type 2 cytokines in bronchoalveolar lavage fluid were measured in vivo. The expression of the aryl hydrocarbon receptor was markedly increased when MnBP was administered. MnBP treatment increased AHR, airway inflammatory cells (including eosinophils), and type 2 cytokines following OVA challenge compared to vehicle‐treated mice. However, apigenin treatment reduced all asthma features, such as AHR, airway inflammation, type 2 cytokines, and the expression of the aryl hydrocarbon receptor in MnBP‐augmented eosinophilic asthma. Our study suggests that MnBP exposure may increase the risk of eosinophilic inflammation, and apigenin treatment may be a potential therapy for asthma exacerbated by endocrine‐disrupting chemicals.

## 1 INTRODUCTION

The prevalence of asthma has been increasing over several decades as lifestyles become westernized world widely.^1^ Although many factors may influence the increased prevalence of asthma, frequent exposure to environmental pollutants and their interaction with host genetics have recently emerged as one of the major causes.^2^ Endocrine‐disrupting chemicals (EDCs) are exogenous chemicals that interfere with the hormonal system of our body, and it has been known to contribute to reproductive and developmental diseases, cancer formation, obesity, and even inflammatory diseases.^3^

Phthalates, one type of EDCs, are mainly used as a plasticizer and are contained in the manufacture of various products in our daily lives, including cosmetics, medical devices, food packaging, children's toys, and polyvinyl chloride plastics. As a result, humans are exposed to phthalates through various routes, including water, air, skin contract, and food. Recently, several epidemiological studies have demonstrated that continuous exposure to phthalates can increase the risk of chronic diseases including cardiovascular diseases and diabetes.^4^, ^5^ Interestingly, it has been reported that exposure to phthalates also increases the risk of allergic asthma. Mono‐benzyl phthalate was reported to be associated with the prevalence of self‐reported asthma in children in the National Health and Nutrition Examination Survey,^6^ and exposure to di‐(2‐ethylhexyl) phthalate (DEHP) and butylbenzyl phthalate after birth has also been reported to be associated with an increase in childhood asthma.^7^ However, it is not yet known which phthalates and which mechanisms contribute to the development of asthma.

The aryl hydrocarbon receptor (AhR) is a cytoplasmic receptor and transcription factor activated by diverse compounds from the environment, diet, and microbiome, and is a key sensor allowing immune cells to adapt to environmental conditions.^6^, ^7^, ^8^ Diesel exhaust particles can activate bronchial epithelial cells via AhR, and these responses can abolish by the knockdown of AhR by siRNA.^9^ Exposure to particulate matter also activates AhR, and this activation is known to induce neutrophil inflammation in a mouse model of asthma.^10^

Interestingly, natural substances may hold potential as therapeutics for artificial EDCs. Flavonoids are polyphenolic metabolites found in vegetables, fruits, and beverages that have antioxidant and immune‐modulating properties through their inhibitory effect on AhR.^11^ The natural flavonoid apigenin has been shown to attenuate particulate matter‐induced asthma in a model through the modulation of interleukin (IL)‐17 and the downregulation of nuclear factor (NF)‐κB expression,^12^ and the flavonoid, quercetin, has been shown to reduce allergic airway inflammation and hyperresponsiveness.^13^ AhR, the receptor for both EDCs and flavonoids, is expressed in all major inflammatory cells in allergic asthma, including eosinophils, T cells, B cells, and epithelial cells.^10^

Therefore, in this study, we aimed to investigate the effects of mono‐n‐butyl phthalate (MnBP) and its antagonists on allergic asthma in an EDC‐aggravated allergic asthma model, and to examine their effects on major inflammatory cells in allergic inflammation.

## 2 MATERIALS AND METHODS

### 2.1 Animals

Female BALB/c mice (6 weeks old; weight, 20 ± 2 g) were obtained from the Jackson Laboratory. The animals were housed under specific pathogen‐free conditions and were maintained on a 12‐h light/dark cycle with food and water provided ad libitum. All animal experiments conducted in this study were approved by the Institutional Animal Care and Use Committee of Ajou University (IACUC‐2021‐0048).

### 2.2 Eosinophilic asthma mouse model, MnBP and apigenin treatment

Mice were sensitized intraperitoneally with 10ug of ovalbumin (OVA) in 1 mg of alum (Imject™ Alum Adjuvant; Thermo Fisher Scientific) on days 0 and 14, as previously described.^14^ On days 28, 29, and 30, mice were subjected to allergen challenges by nebulization with 1% OVA for 30 min, using an ultrasonic nebulizer (NE‐Y2, Omron). In some groups, mice were given 3 mg/mL of MnBP was administered through drinking water on days 0–30 with or without 25 mg/kg of apigenin (Sigma‐Aldrich) treatment orally from day 14 to day 28. Control animals received PBS only. After 48 h of the last OVA challenge, mice were sacrificed for further analysis.

### 2.3 Evaluation of airway resistance, cytokines in bronchoalveolar lavage fluid and lung histology

Airway resistance was measured using the FlexiVent system (Scireq). On day 32, mice were anaesthetised with pentobarbital sodium and intubated with a cannula. After being connected to a computer‐controlled small‐animal ventilator, the mice were ventilated with a tidal volume of 10 mL/kg at a frequency of 150 breaths/min. The baseline airway resistance (R_L_) of each mouse was recorded. Subsequently, a dilution series of acetyl‐β‐methylcholine chloride (MCh) from 3.12 to 25 mg/mL were gradually introduced to the mice, and the R_L_ values at each concentration were recorded. After measuring airway resistance, bronchoalveolar lavage (BAL) fluid was harvested with a wash of 1 mL of phosphate‐buffered saline (PBS) plus 2% bovine serum albumin (Sigma‐Aldrich). After the BAL fluid was centrifuged at 1500 rpm for 5 min at 4°C, leukocytes were quantified with a haemocytometer, and differential cell counts were performed by counting at least 200 cells on cytospin slides stained with Wright–Giemsa stain. The supernatant was collected and stored at −70°C until further analysis. The levels of IL‐4, IL‐5, IL‐13 (eBioscience), in the BAL fluid were measured by enzyme‐linked immunosorbent assay (ELISA) according to the manufacturer's instructions. Fixed tissues were sectioned at 5‐μm thickness, and lung tissues were stained with Haemotoxylin and Eosin (H & E) for the evaluation inflammatory cell infiltration and Periodic Acid Schiff (PAS) for the evaluation of mucus production. The number of inflammatory cells per μm^2^ of perivascular and peribronchial areas and the number of mucus‐containing cells per μm^2^ of basement membrane were determined by ImageJ (National Institutes of Health).

### 2.4 Western blotting to detect aryl hydrocarbon receptor and eosinophilic granular proteins

Thirty‐five micrograms of protein were isolated from each tissue homogenate using RIPA buffer. Proteins was separated 12% SDS‐PAGE gel and transferred onto polyvinylidene difluoride (PVDF) membranes (Bio‐Rad Laboratories, Inc.). Blocking in 5% skim milk (Sigma‐Aldrich) in Tris buffered saline containing 0.01% Tween 20 (TBST‐T) for 1 h at room temperature. The membranes were then incubated with primary antibodies against aryl hydrocarbon receptor (sc‐133,088; Bioss antibodies), Arnt (sc‐17,811; Santa Cruz Biotechnology), CYP1A1 (MBS9607535; MyBioSource), CYP1B1 (MBS9607056; MyBioSource), EPX (bs‐3881R; Bioss antibodies), EPO (bs‐8615R; Bioss antibodies), and β‐actin (sc‐47,778; Santa Cruz Biotechnology) After washing extensively in Tween‐TBS, the membranes were incubated with biotinylated secondary antibodies for 1 h at room temperature. Antibody binding was visualized using an ECL detection kit (GE Healthcare), and images were acquired using a gel doc system (Bio‐Rad Laboratories, Inc.).

### 2.5 cDNA preparation and real time‐polymerase chain reaction

Total RNA was isolated from mouse lung tissue using the RNeasy Mini Kit (Qiagen). Single‐stranded cDNA was then synthesized from 0.5 μg of total RNA using iScript cDNA synthesis kit (Bio‐Rad Laboratories, Inc.). The primers sequences were as follows: AhR, forward, 5‐AGCTTCACTGGGCTCTAAAC‐3, and reverse, 5‐GAATTATCCAGCAGGCACCT‐3. cDNA was quantified by means of real time‐polymerase chian reaction (RT‐PCR) using Power SYBR Green Master Mix (Thermo Fisher Scientific). Data were analysed through QuantStudio 5 software (Thermo Fisher Scientific). The expression of cytokine genes in bronchial tissue was normalized to GAPDH expression.

### 2.6 Mononuclear cell isolation and ex vivo cytokines measurement

Mononuclear cells (MNC) from OVA‐sensitized and ‐challenged mice were isolated as previously described.^15^ Briefly, spleen cells from sensitized and challenged mice were harvested by mincing the tissues and passing them through a stainless‐steel sieve. After washing with PBS, MNCs were isolated by histopaque gradient centrifugation (Sigma‐Aldrich). Then, spleen MNCs (1 × 10^6^ cells per well) were cultured for 24 h with OVA (10 μg/mL) and OVA + MnBP (10 μM) or apigenin (1 μM). Culture supernatants were evaluated to measure type 2 cytokines as described above.

### 2.7 In vitro cells preparation and culture

Human lung epithelial A549 cells were purchased from ATCC and maintained at 37°C in a humidified incubator containing 5% CO_2_ in RPMI medium with 10% fetal bovine serum (Thermo Fisher Scientific), supplemented with 100 U/mL penicillin and 100 μg/mL streptomycin (Thermo Fisher Scientific). The cells were seeded onto culture plates and allowed to adhere for several hours. They were then stimulated with 1 μM phorbol‐12‐myristat‐13‐acetate (PMA) (Sigma‐Aldrich) in 1× PBS with various compounds. The cytokines released into the supernatant were quantified using IL‐25 and IL‐33 (R & D System) by ELISA. The human eosinophilic cell line Eol‐1 was grown in RPMI 1640 medium, supplemented with 10% heat‐inactivated FBS, 2 mM L‐glutamine, 100 U/mL penicillin, and 100 μg/mL streptomycin in a humidified atmosphere with 5% CO_2_ at 37°C. Eol‐1 cells were maintained at of 1 × 10^5^ cells per well. For the cytokine stimulation study, Eol‐1 cells were incubated with 0.5 μM butyric acid (Sigma‐Aldrich), or with MnBP (5 μM) and apigenin (1 μM) for 24 h. Culture supernatants were used for evaluating IL‐5 and eosinophil cationic protein (ECP) (MyBioSource) measured by ELISA. Jurkat T cells (clone E6‐1, TIB‐152; LGC Promochem) were routinely maintained in RPMI‐1640 medium containing 10% fetal bovine serum, 1% L‐glutamine (all from Biochrom AG), 1% penicillin (100 U/mL)/streptomycin (100 mg/mL) (PAA) at an atmosphere of 5% CO_2_ and 95% humidity at 37°C in a CO_2_ incubator. Jurkat T cells were seeded at a density of 1 × 10^5^ cells per well. Cell stimulation was induced by PMA (1 μM), MnBP (10 μM) and apigenin (1 μM) for 24 h. Culture supernatants were used for evaluating type 2 cytokines.

### 2.8 A549 cell by siRNA transfection

Human lung epithelial A549 cells (5 × 10^5^ cells per well) were transfected with siRNA against AhR (siAhR) or scramble siRNA (negative control for AhR siRNA) (Thermo Fisher Scientific) using Lipofectamine RNAiMAX reagent (Invitrogen) for 24 h, and cells were then stimulated with MnBP and apigenin at different concentrations and time intervals.

### 2.9 Statistical analysis

The differences between treatment groups were assessed using one‐way analysis of variance (anova) and Tukey's post hoc test, unless indicated otherwise. The differences between the stimulated and control cells in the in vitro assay were assessed using the Wilcoxon signed‐rank test. All statistical analyses were performed using SPSS software version 23.0 (SPSS Inc.), and a *p*‐value of <0.05 was considered significant.

## 3 RESULTS

### 3.1 The expression of aryl hydrocarbon receptor in MnBP‐treated allergic asthma model

**FIGURE 1:**
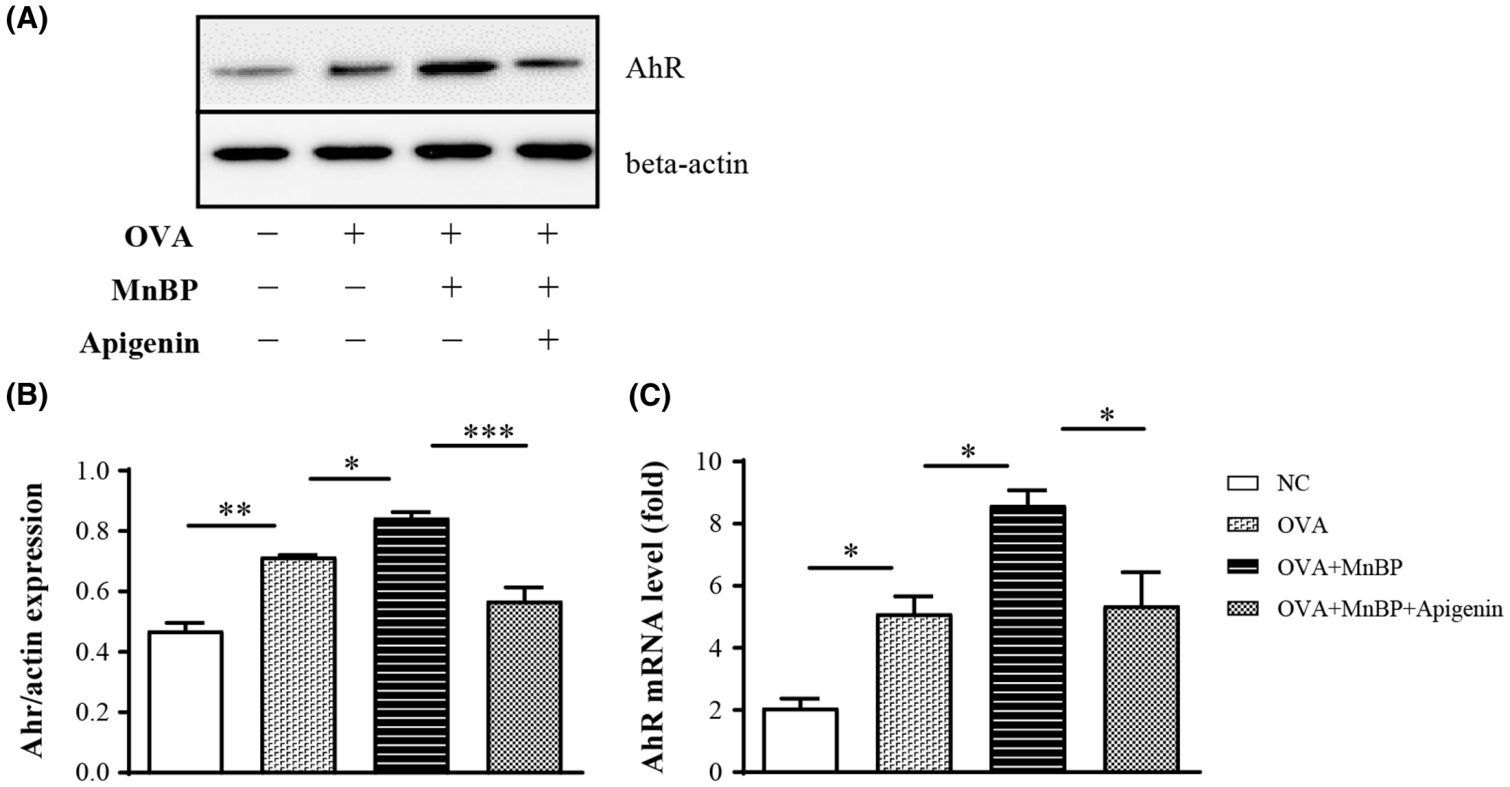
Protein and mRNA expression of aryl hydrocarbon receptor (A) Western blotting showed the protein expression of AhR and beta‐actin in lung tissue from MnBP and apigenin treated mice. (B) Quantitative data were obtained by densitometric analysis for the expression of AhR relative to β‐actin. (C) mRNA levels of AhR were evaluated in lung tissue from MnBP and apigenin treated mice by qRT‐PCR. Expression was defined by fold change in relative to control. Data represent mean ± SEMs of three independent experiments. *p < 0.05, **p < 0.01 and ***p < 0.001.

First, the expressions of aryl hydrocarbon receptor known as a receptor of phthalate and flavonoid were measured by western blot in lung tissue of our MnBP‐treated allergic asthma model. The expression of aryl hydrocarbon receptor was markedly increased following OVA challenge in OVA‐sensitized mice compared to negative control mice, and this expression was significantly augmented when MnBP was exposed in the same mice group. In addition, apigenin administration effectively reduced the expression of aryl hydrocarbon receptor in MnBP‐treated allergic asthma mice (Figure 1A,B). The mRNA levels of aryl hydrocarbon receptor were also investigated by RT‐PCR. Same as the above result, the mRNA level of aryl hydrocarbon receptor was increased by MnBP exposure and decreased by apigenin administration in OVA‐sensitized and challenged mice (Figure 1C).

### 3.2 The effects of MnBP and apigenin in allergic asthma mouse

**FIGURE 2:**
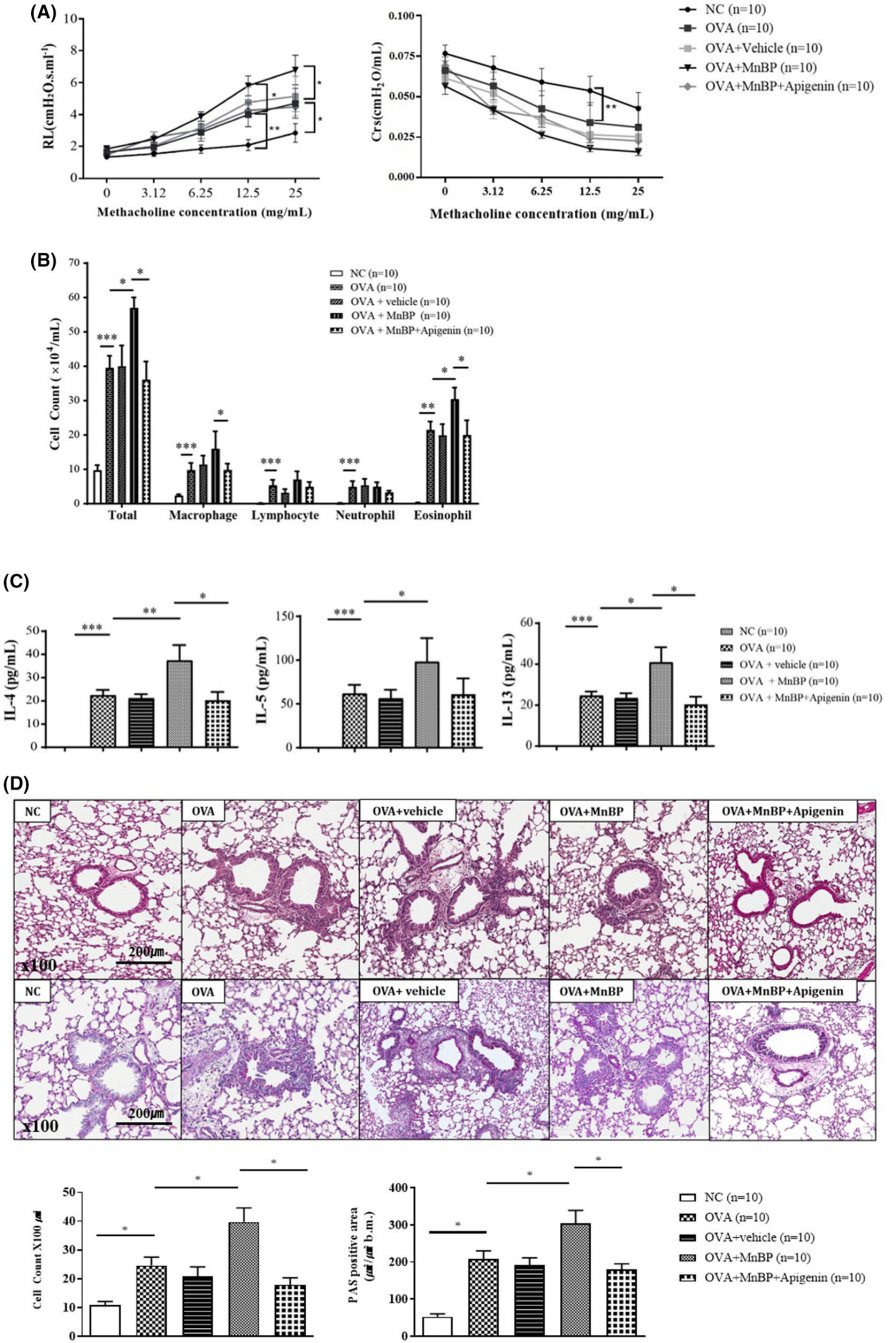
The effects of MnBP and apigenin on airway hyperresponsiveness and inflammation (A) The effects of MnBP and apigenin on airway hyperresponsiveness. (B) Total and differential cell counts in the BALF. (C) The level of type 2 cytokines in the BALF was measured by ELISA. (D) The lung tissues were stained with H & E stain for inflammation cells and with PAS stain for mucus‐containing cells. Quantitative data were obtained by ImageJ program for H & E and PAS stain. Data were shown as means ± standard errors. (*n*=10) *p < 0.05, **p < 0.01 and ***p < 0.001.

We evaluated airway inflammation and airway hyperresponsiveness (AHR) after the administration of MnBP or apigenin in OVA‐sensitized and challenged mice. MnBP administration significantly increased AHR and airway inflammatory cells which were total cells, macrophages, and eosinophils compared to those of OVA‐sensitized and challenged mice (Figure 2A,B). The levels of type 2 cytokines such as IL‐4, IL‐5, and IL‐13 were also increased in MnBP administrated asthmatic mice than in OVA‐sensitized and challenged mice (Figure 2C). In lung tissue, histopathological examination using Haemotoxylin and Eosin and PAS staining showed peri‐bronchial and peri‐vascular inflammatory cell infiltration in OVA‐sensitized and challenged mice, and these features were significantly aggravated after the MnBP exposed mice (Figure 2D). Interestingly, when apigenin was treated in the same allergic asthma model, all asthma features such as AHR, airway inflammation, and type 2 cytokine were statistically significantly reduced compared to when MnBP was administered (Figure 2A–D).

### 3.3 The effects of MnBP and apigenin on eosinophilic activation in allergic mouse model

**FIGURE 3:**
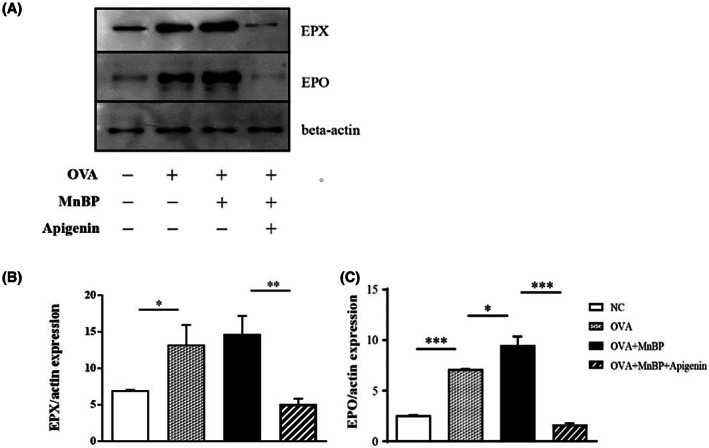
The effects of MnBP and apigenin on eosinophilic granular protein in the lung tissues (A) Western blotting showed the protein expression of EPX, EPO and beta‐actin in the lung tissues from MnBP and apigenin‐treated mice. Quantitative data were obtained by densitometric analysis for the expression of (B) EPX and (C) EPO relative to β‐actin. Data represent mean ± SEMs of two independent experiments. *p < 0.05, **p < 0.01 and ***p < 0.001.

Since eosinophils are the most important effector cells in our allergic asthma model, we measured EPX and EPO, the granular proteins secreted when eosinophils are activated in the lung tissue by western blot. The expressions of EPX and EPO were markedly increased following OVA challenges in OVA‐sensitized mice compared to negative control mice, and these results were significantly augmented by MnBP administration. In line with the above result about the decrease in eosinophilic count in BAL fluid, it was confirmed that the expression of EPX and EPO was significantly reduced when the mice were treated with apigenin (Figure 3).

### 3.4 Ex vivo effects of MnBP and apigenin on type 2 cytokine productions in splenic MNCs

**FIGURE 4:**
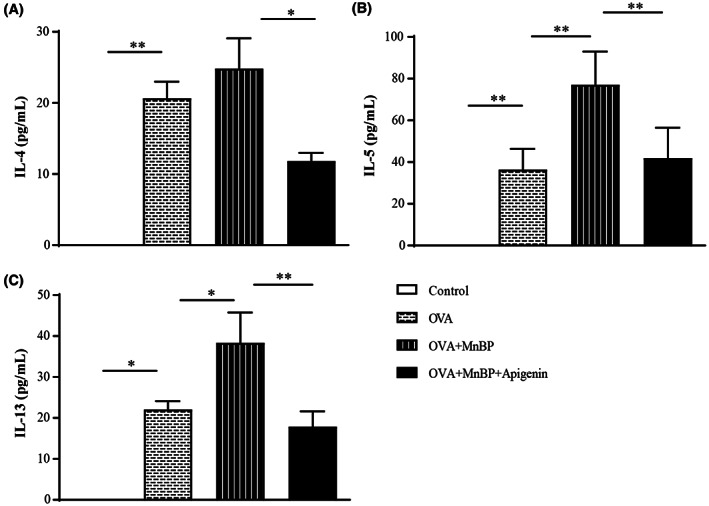
The effects of MnBP and apigenin on type 2 cytokine in the splenic MNCs (A) IL‐4, (B) IL‐5 and (C) IL‐13 levels in supernatants from cultured spleen MNCs isolated from OVA‐sensitized and challenged mice in the presence or absence of OVA (10 μg/mL) and the level of MnBP (10 μM) or apigenin (1 μM) determined by ELISA. The data are expressed as the mean ± SD from three independent experiments. *p < 0.05 and **p < 0.01.

Next, the results of type 2 cytokines were confirmed again in ex vivo splenic MNCs. OVA stimulation for 24 h significantly produced type 2 cytokines in splenic MNCs of OVA‐sensitized and challenged mice, and the levels of IL‐4, IL‐5, and IL‐13 were significantly increased by MnBP treatment and decreased by apigenin administration in the same cell groups (Figure 4).

### 3.5 In vitro effects of MnBP and apigenin on type 2 cytokine productions using Jurkat T cells

**FIGURE 5:**
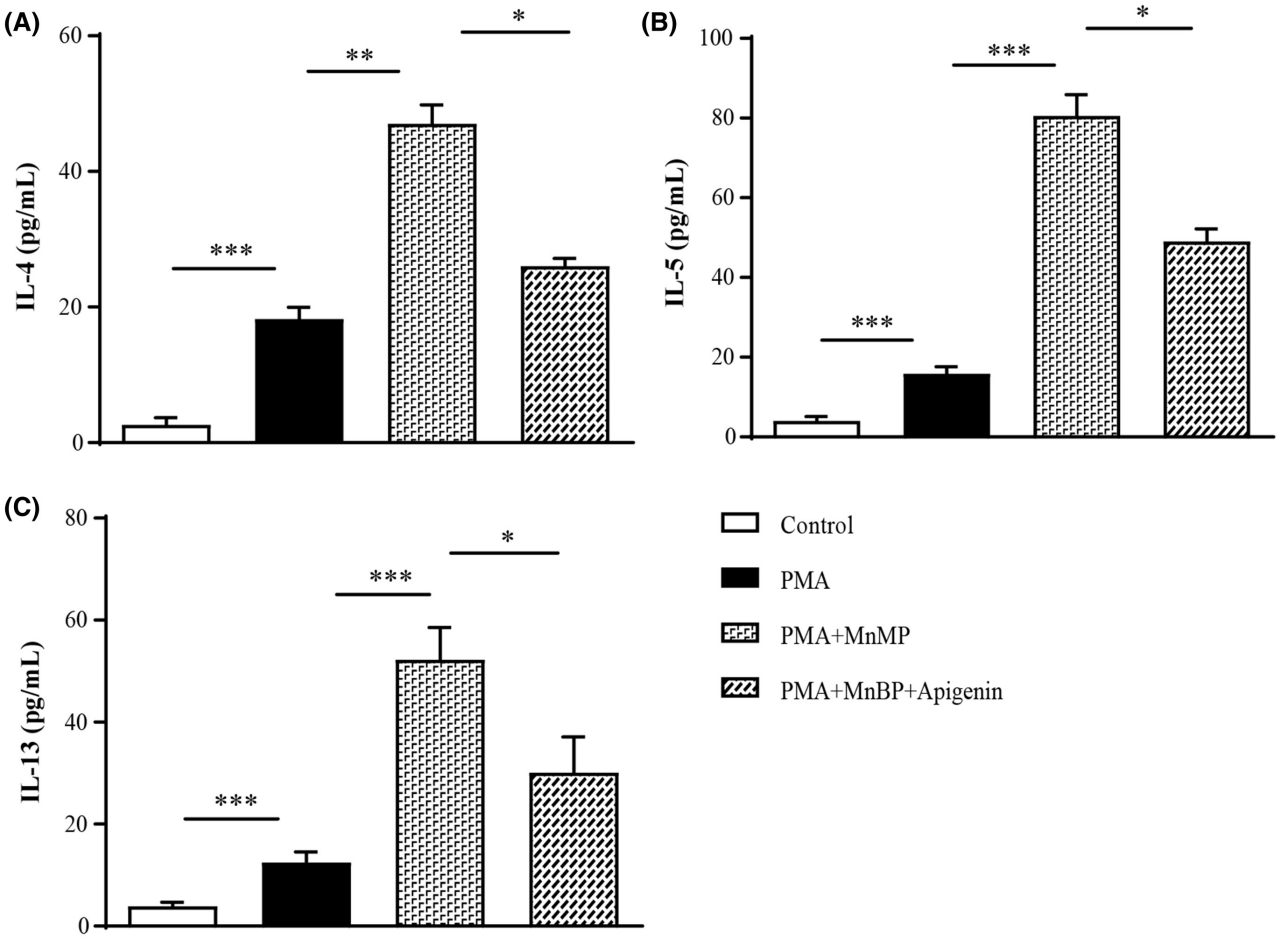
The effects of MnBP and apigenin on type 2 cytokines in Jurkat T cells Jurkat T cells were activated with PMA (1 μM) in combination with MnBP (10 μM) and apigenin (1 μM). (A) IL‐4, (B) IL‐5 and (C) IL‐13 levels in supernatants from Jurkat T cell were determined by ELISA. The data are expressed as the mean ± SD from three independent experiments. *p < 0.05, **p < 0.01 and ***p < 0.001.

The cytotoxicity after MnBP or apigenin treatment in Jurkat T cells was measured by CCK8 assay. As shown in Figure S1, each MnBP and apigenin concentration up to 100 μM had no effect on cell viability. As with the in vivo and ex vivo results, PMA alone treatment increased the secreted levels of IL‐4, IL‐5, and IL‐13 compared to negative control. These secretions were further enhanced by MnBP treatment and effectively reduced by apigenin treatment (Figure 5).

### 3.6 The effects of MnBP and apigenin on eosinophilic activation in Eol‐1 cells

**FIGURE 6:**
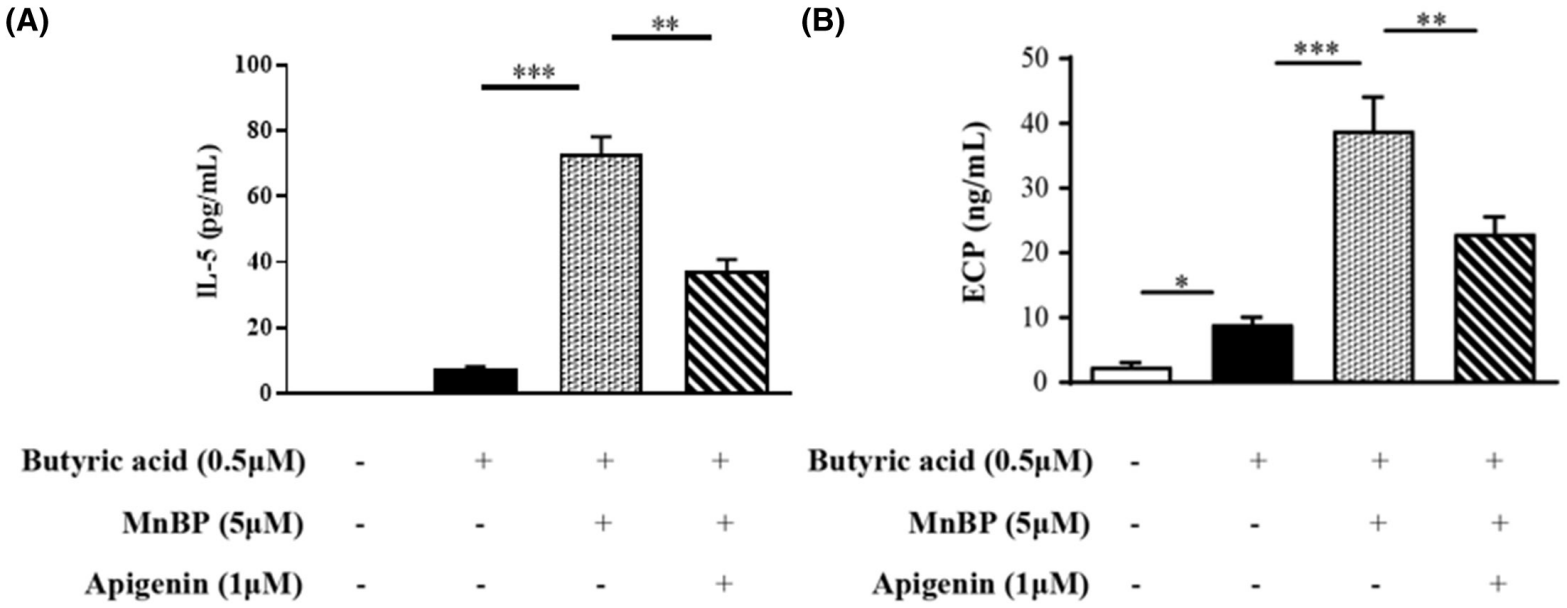
Cytokine production in Eol‐1 cells with MnBP and apigenin Eol‐1 cells were pre‐treated with butyric acid (5 μM) for 30 min and incubated for 24 h with MnBP (5 μM) and apigenin (1 μM). (A) IL‐5 and (B) ECP released into the supernatant quantified using ELISA. The data are expressed as the mean ± SD from three independent experiments. *p < 0.05, **p < 0.01 and ***p < 0.001.

The effects of MnBP and apigenin on eosinophil activity were re‐evaluated in EoL‐1 cells in vitro. As shown in Figure 6, MnBP administration augmented the secretions of IL‐5 and ECP compared to that of butyric acid stimulation only, and AhR receptor antagonist, apigenin, treatment effectively decreased those levels.

### 3.7 The inhibitory effects of aryl hydrocarbon receptor using siRNA and its antagonist in vitro epithelial cells

**FIGURE 7:**
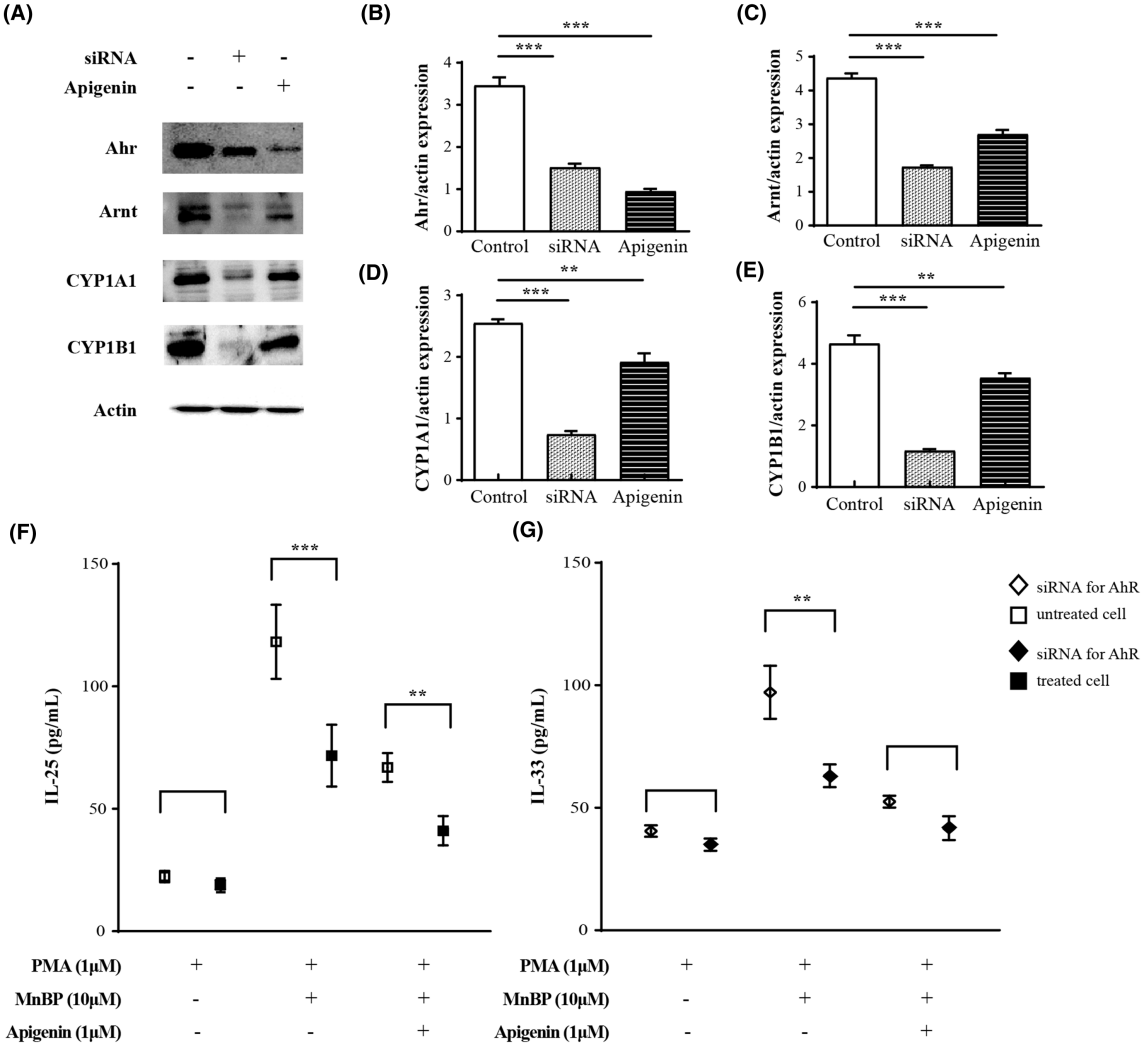
The effects of AhR siRNA on human lung epithelial cells (A) The expression levels of AhR, Arnt, CYP1A1 and CYP1B1 were determined in human lung epithelial cells. A549 cells were subject to western blot with a primary antibody against AhR, Arnt, CYP1A1 and CYP1B1. β‐actin protein was used as an internal control. Quantitative data were obtained by densitometric analysis for the expression of (B) AhR, (C) Arnt, (D) CYP1A1 and (E) CYP1B1 relative to β‐actin. (F) IL‐33 and (G) IL‐25 levels in A549 cells before and after transfection with siRNA for AhR. Cytokine expression in A549 cells treated with MnBP and apigenin. The data are expressed as the mean ± SD from three independent experiments. **p < 0.01 and ***p < 0.001 between the cell after transfection with siRNA for AhR and without siRNA for AhR.

Finally, the inhibitory effects of siRNA for AhR and its antagonist on epithelial cells were evaluated because of airway epitheliums are the first contact cells when exposed to phthalates and they can orchestrate airway inflammation in the pathogenesis of asthma. When A549 cells were transfected with siRNA for AhR or treated with apigenin, the protein expressions of AhR and its nuclear translocator (ARNT) were decreased compared to the not treated negative control (Figure 7A–D). Next, the epithelial‐producing cytokines IL‐25 and IL‐33 were measured after siRNA for AhR treatment (Figure 7F,G). Compared to siRNA non‐transfected epithelial cells, siRNA‐ transfected cells produced low levels of IL‐25 and IL‐33. In addition, these effects were significantly increased when combined with apigenin treatment in siRNA‐transfected cells.

## 4 DISCUSSION

In this study, we demonstrated that MnBP could exacerbate pre‐existing asthma using an eosinophilic asthma model. Asthma is frequently exacerbated by various factors such as infection, allergens, and environmental pollutants. In recent studies, EDCs have been identified as a potential factor that can cause asthma exacerbation.^16^, ^17^ High molecular weight phthalate metabolites were positively correlated with allergic symptoms in a large population‐based cross‐sectional study^18^ and significantly increased level of benzylbutyl phthalate was found to be the potential risk factor for allergic asthma.^19^ In our previous study, we found that MnBP could play a pivotal role in the exacerbation of eosinophilic asthma through the regulation of the nuclear factor erythroid 2‐related factor 2 and nuclear factor kappa B pathways.^20^ In this study, we also confirmed that airway hyperresponsiveness and airway inflammation were significantly increased in MnBP‐treated mice compared to OVA‐sensitized and challenged mice that were not treated with MnBP. In addition, we evaluated the mechanisms by which MnBP exacerbates eosinophilic asthma and the effects of potential therapeutic agents based on these mechanisms.

AhR is a highly evolutionarily conserved ligand‐activated transcription factor that functions as a systemic environmental sensor for a variety of ligands, including environmental, dietary, microbial, and xenobiotic substances^10^ and is expressed in most immune cells such as dendritic cell, mast cell, lymphocytes, and eosinophil.^21^ When bound to ligands, AhR undergoes a conformational change, translocates from the cytosol to the nucleus, and dimerizes with AhR nuclear translocator (Arnt). The AhR/Arnt heterodimer then binds to the xenobiotic responsive element in the nucleus, leading to a range of toxicological effects.^22^ As a receptor for EDCs and present on key immune cells in asthma, AhR is of great interest in MnBP‐induced asthma. In this study, we found that the protein expression and mRNA level of AhR were elevated in the lung tissue when MnBP was administered with OVA. Although not using EDCs, recent studies using other pollutants such as PM2.5 or diesel exhaust particles showed that airway inflammation in asthma aggravated through AhR mediation.^9^, ^23^ In addition, these results were significantly reduced in AhR knockout mice.^24^ Taken together, AhR is the most important mediator of phthalates, suggesting that it could be a therapeutic target for phthalate‐aggravated asthma.

Since AhR contributed to MnBP‐induced asthma exacerbation, we investigated the effect of an AhR antagonist on our MnBP‐treated allergic asthma model. Flavonoids are polyphenolic plant secondary metabolites with potent antioxidant, anti‐inflammatory, and immune‐modulating effects.^25^ Recent studies have shown that they can modulate immune functions by binding to AhR.^26^, ^27^ Flavonoids such as apigenin, quercetin, and kaempferol showed marked inhibitory effects on AhR activation in an in vitro bioassay.^28^ and can also act as regulators of the differentiation of naïve CD4+ T cells into effector T cell subsets.^29^, ^30^ We used apigenin as a potential candidate drug in our MnBP‐treated eosinophilic asthma model. Apigenin is a naturally occurring plant flavone that has already been shown to have therapeutic effects on the reduction of type 2 cytokines, eosinophilic activation, and allergic specific IgE production.^31^, ^32^ As expected, apigenin treatment reduced the expression of AhR in EDC‐augmented eosinophilic asthma mice and significantly decreased airway inflammation, type 2 cytokines, and airway hyperresponsiveness compared to mice that were not treated with apigenin. To our knowledge, this study is the first to confirm the therapeutic effect of flavonoids in an EDC‐augmented eosinophilic asthma model.

Since allergic inflammation is a complex orchestration of immune cells such as eosinophils, epithelial cells, and T lymphocytes, we evaluated the potential of apigenin as a new drug candidate in an in vitro cell study. Therefore, we evaluated the therapeutic effect of apigenin on these cells in the order in which they are affected by aggravating factors such as EDCs in the development of allergic inflammation. First, apigenin administration reduced the expression of AhR and its nuclear translocator, ARNT, in epithelium and decreased the secretion of IL‐25 and IL‐33 which can affect the downstream pathway of allergic inflammation. These results were confirmed again with siRNA for AhR transfected epithelial cells, and it was also shown that the secretion of IL‐25 and IL‐33 was synergistically decreased when apigenin and siRNA were treated together. Although previous studies have shown that flavonoids such as apigenin inhibit pro‐inflammatory NF‐kappa B, IRF, and Akt signalling in epithelial cells,^33^ this is the first study to show reduced IL‐25 and IL‐33 levels after apigenin treatment. Type 2 T cells and their cytokines are typical hallmarks of allergic inflammation. Because increased type 2 cytokines were detected in MnBP‐treated mice compared to those in MnBP‐untreated allergic asthma mice, we confirmed these phenomena through in vitro experiments. Splenic MNCs and Jurkat T cells showed quite similar patterns to those described above as expected. These results are in line with the previous studies.^32^, ^34^ However, these studies only showed the effects of flavonoids in a normally OVA‐stimulated allergic asthma model. The difference in this study is that it demonstrated the effectiveness of flavonoids in asthma exacerbated by MnBP, and this result may show promise as a therapeutic agent for flavonoids in asthma exacerbated by EDCs.

In this study, apigenin was used to evaluate the role in eosinophils but other flavonoids have already been shown to play protective roles in eosinophilic inflammation in previous studies.^35^, ^36^, ^37^ For example, Flavonoid‐like chemical, wogonin, induced eosinophilic apoptosis and attenuated the number of eosinophils in the lung^36^ and this flavonoid also decreased eosinophilic nasal polyp formation in chronic rhinosinusitis murine model by suppressing HIF‐1α and surviving expression.^35^ Since eosinophilic activation is more important than the number of eosinophils in allergic inflammation, our study measured activation markers EPX, EPO and ECP in addition to the number of eosinophils. We found elevated eosinophilic marker levels after administration of MnBP and reduced those levels after treatment with apigenin in the asthma mouse model and in vitro Eol‐1 cells.

Although this study has the fundamental disadvantage that it is based on a mouse asthma model, we tried to show the effects of MnBP and apigenin using human Jurkat T cells and human EoL‐1 eosinophils cell‐line. Flavonoids such as apigenin are found in many foods and are known to be non‐harmful to humans,^38^ so the next study should be conducted on human and human samples. In addition, since our previous study showed that phthalate can exacerbate urticaria,^39^ this study can be extended to other allergic diseases besides asthma. In summary, our study demonstrated that that MnBP exposure can exacerbate allergic asthma and apigenin treatment can be a potential therapeutic for endocrine disrupting chemical‐aggravated asthma through the decreased activation of epithelial cells, T cells, and eosinophils.

## AUTHOR CONTRIBUTIONS

**Seo‐Hee Kim:** Data curation (lead); formal analysis (lead); investigation (lead); methodology (supporting); writing – original draft (lead); writing – review and editing (supporting). **Quang Luu Quoc:** Data curation (supporting); formal analysis (supporting); investigation (supporting); methodology (supporting). **Hae‐Sim Park:** Funding acquisition (lead); supervision (supporting). **Yoo Seob Shin:** Conceptualization (lead); methodology (lead); supervision (lead); writing – review and editing (supporting).

## CONFLICT OF INTEREST STATEMENT

The authors confirm that there is no conflict of interest.

## Supporting information


Figure S1.

## Data Availability

The data that support the findings of this study are available on request from the corresponding author. The data are not publicly available due to privacy or ethical restrictions.
