# Supplementary figures and images for: The effect of apigenin, an aryl hydrocarbon receptor antagonist, in Phthalate‐Exacerbated eosinophilic asthma model
Source: J Cell Mol Med. 2023 Jun 14;27(13):1900–10. doi: 10.1111/jcmm.17804 (PMC10315829; doi:10.1111/jcmm.17804)

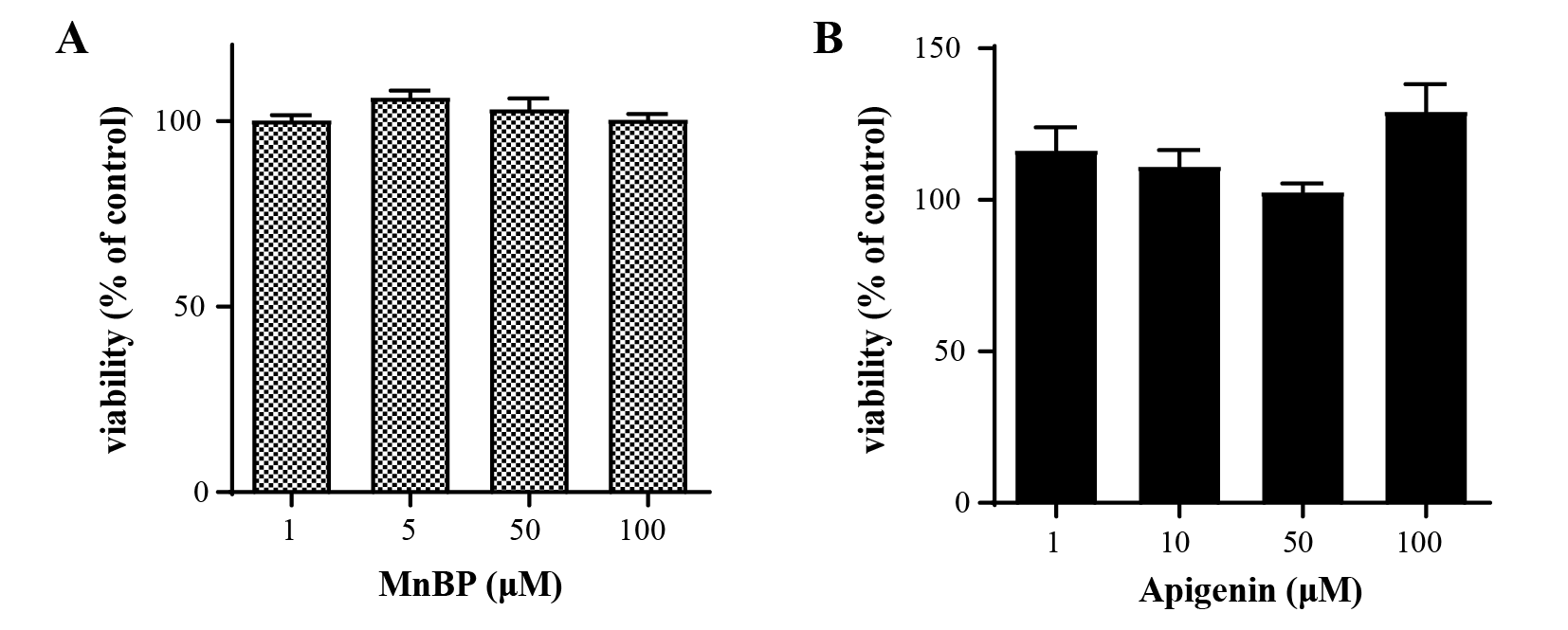

Supplement: Supplementary file 1 — Figure S1. [file JCMM-27-1900-s001.tif]
